# Assessment of circulating biomarkers for potential pharmacodynamic utility in patients with lymphoma

**DOI:** 10.1038/sj.bjc.6606082

**Published:** 2011-01-18

**Authors:** A Greystoke, J P B O'Connor, K Linton, M B Taylor, J Cummings, T Ward, F Maders, A Hughes, M Ranson, T M Illidge, J Radford, C Dive

**Affiliations:** 1 Clinical and Experimental Pharmacology Group, The Paterson Institute for Cancer Research, The University of Manchester, Wilmslow Road, Withington, Manchester M20 4BX, UK; 2 School of Cancer, Enabling Sciences and Technology, The University of Manchester, Manchester M20 4BX, UK; 3 Department of Medical Oncology, Cancer Research UK, Manchester M20 4BX, UK; 4 Manchester Academic Health Science Centre, Manchester M20 4BX, UK; 5 Department of Clinical Radiology, The Christie NHS Foundation Trust, Manchester M20 4BX, UK

**Keywords:** Hodgkin and non-Hodgkin lymphoma, biomarkers, nucleosomal DNA, cytokeratin 18, FLT3 ligand

## Abstract

**Purpose:**

Treatment efficacy and toxicity are difficult to predict in lymphoma patients. In this study, the utility of circulating biomarkers in predicting and/or monitoring treatment efficacy/toxicity were investigated.

**Patients and methods:**

Circulating biomarkers of cell death (nucleosomal DNA (nDNA) and cytokeratin 18 (CK18)), and circulating FLT3 ligand, a potential biomarker of myelosuppression, were assessed before and serially after standard chemotherapy in 49 patients with Hodgkin and non-Hodgkin lymphoma. Cytokeratin 18 is not expressed in lymphoma cells so is a potential biomarker of epithelial toxicity in this setting. Tumour response was assessed before and after completion of chemotherapy by 2D and 3D computed tomography radiological response.

**Results:**

Baseline nDNA level was significantly higher in all lymphoma subtypes compared with 61 healthy controls and was prognostic for progression-free survival in diffuse large B-cell lymphoma (DLBCL). Decreases in nDNA levels were observed in the first week after chemotherapy; in FL, early falls in nDNA predicted for long remission following therapy. In DLBCL, elevations in nDNA occurred in cases with progressive disease. Circulating CK18 increased within 48 h of chemotherapy and was significantly higher in patients experiencing epithelial toxicity graded >3 by Common Terminology for Classification of Adverse Events criteria. FLT3 ligand was elevated within 3–8 days of chemotherapy initiation and predicted those patients who subsequently developed neutropenic sepsis.

**Conclusion:**

These data suggest circulating biomarkers contribute useful information regarding tumour response and toxicity in patients receiving standard chemotherapy and have potential utility in the development of individualised treatment approaches in lymphoma. These biomarkers are now being tested within multicentre phase III trials to progress their qualification.

The majority of lymphomas are treatable and many, with modern treatment approaches are potentially curable. There is, however, a need to mitigate the risk of life-threatening treatment toxicity and to overcome the poor prognosis associated with treatment failure. This has led to interest in the development of biomarkers that report the efficacy/toxicity of treatments and inform the development of individualised treatment strategies. Tissue-based biomarkers are the traditional ‘gold standards’ used in drug development, but circulating biomarker analyses are more readily applicable to most clinical settings. A biomarker detected in the blood and measured serially could provide a real-time assessment of a patient's disease course and response to treatment (Workman *et al*, 2006). Here, as a first step towards biomarker qualification, a panel of circulating biomarkers of cell death and myelosuppression has been evaluated for its potential to predict and/or report drug efficacy and toxicity in patients with lymphoma receiving standard chemotherapies.

During apoptosis, chromatin is cleaved into nucleosomal strands by caspase-activated endonucleases (Oberhammer *et al*, 1993). Nucleosomes reside in membrane-bound apoptotic fragments, undergo macrophage phagocytosis and are released into the blood as nucleosomal DNA (nDNA) (Jiang *et al*, 2003). Circulating nDNA levels are elevated in patients with cancer including lymphoma (Deligezer *et al*, 2006) compared with healthy controls, and have demonstrated prognostic and pharmacodynamic utility in studies of lung cancer and other tumour types (Holdenrieder *et al*, 2008; Hou *et al*, 2009). In this study of nDNA in patients with lymphoma, we postulated that baseline nDNA predicts treatment outcome and that changes in nDNA levels during therapy act as pharmacodynamic and surrogate biomarkers of response.

Cytokeratin 18 (CK18), a major component of an epithelial cell cytoskeleton, is not expressed in cells of lymphoid origin. Consequently, elevated circulating CK18 levels reflect epithelial damage as reported in several non-malignant conditions including sepsis, hepatitis and ischaemic heart disease (Adlbrecht *et al*, 2007; Feldstein *et al*, 2009; Hofer *et al*, 2009). Here, we postulated that circulating CK18 levels measured soon after initiating treatment for lymphoma would be useful for monitoring and/or predicting subsequent epithelial toxicity.

Myelosuppression and potentially life-threatening sepsis are common complications of chemotherapy (Kuderer *et al*, 2007). FLT3 ligand, a cytokine that binds the FLT3 receptor, acts synergistically with other cytokines such as stem cell factor and granulocyte colony-stimulating factor in the maintenance of the stem cell compartment and in progenitor expansion/differentiation during haematopoiesis. FLT3 ligand may be particularly important in expansion of the stem cell compartment in response to bone marrow stress (Buza-Vidas *et al*, 2007). In this study, we postulated that changes in soluble FLT3 ligand in patients receiving standard doses of chemotherapy would provide an early warning of severe myelosuppression that could in future be used to guide early intervention with growth factors.

In this study, we have evaluated the utility of nDNA to monitor treatment response, CK18 to monitor and/or predict epithelial toxicity and FLT3 ligand to monitor myelotoxicity in patients with lymphoma receiving standard chemotherapy. This study was also designed with a view to exploring the potential utility of these circulating biomarkers in future clinical trials investigating individualised treatment strategies.

## Patients and Methods

### Patients and blood sample collection

The study received ethical approval from the North Manchester Research Ethics Committee in December 2006 and was conducted according to good clinical practice. Patients with a history of auto-immune disease, viral hepatitis or HIV were excluded as these conditions are known to be associated with high levels of the biomarkers under investigation. Fifty patients with histologically confirmed Hodgkin (HL), follicular (FL) or diffuse large B-cell lymphoma (DLBCL) were recruited between 2007 and 2009 before starting chemotherapy. One patient found to be ineligible after consent was withdrawn. The clinical characteristics of the remaining 49 patients are shown in Table 1. Patients had standard clinical follow-up (median 479 days (range 10–748 days)).

**Figure 1:**
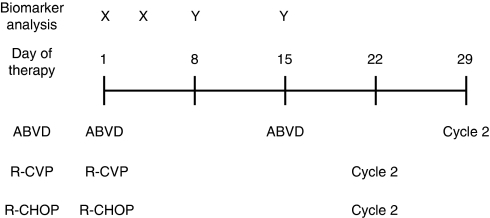
Schedule of administration of anticancer agents and biomarker analysis. X=analysis performed every cycle, Y=analysis performed first cycle only (analysis on treatment days performed on samples taken before therapy); ABVD, doxorubicin 25 mg m^–2^, bleomycin 10 000 IU, vinblastine 6 mg m^–2^ and dacarbazine 375 mg m^–2^ every 14 days. R-CVP, rituximab 375 mg m^–2^, cyclophosphamide 750 mg m^–2^, vincristine 2 mg and prednisone 40 mg m^–2^ (for 5days) every 21 days. R-CHOP, rituximab 375 mg m^–2^, cyclophosphamide 750 mg m^–2^, vincristine 2 mg, doxorubicin 50 mg m^–2^ and prednisone 40 mg m^–2^ (for 5 days) every 21 days.

Control samples were obtained from 61 healthy volunteers at a single time-point. Blood samples were collected from lymphoma patients on days 1, 3, 8 and 15 of the first treatment cycle and days 1 and 3 of subsequent cycles of chemotherapy (Figure 1). On treatment days, samples were taken immediately before the administration of chemotherapy. All samples for serum were processed within 2 h of collection as previously described and all samples analysed within 3 months of collection. These conditions have been shown not to have any significant effect on CK18 levels (Greystoke *et al*, 2008).

### Treatment

Patients were treated with standard chemotherapy according to histological sub-type (Figure 1). Patients with HL were treated with ABVD (doxorubicin 25 mg m^–2^, bleomycin 10 000 IU, vinblastine 6 mg m^–2^ and dacarbazine 375 mg m^–2^) every 14 days. Patients with FL were treated with R-CVP (rituximab 375 mg m^–2^, cyclophosphamide 750 mg m^–2^, vincristine 2 mg and prednisone 40 mg m^–2^ (for 5 days)) every 21 days; in the event of insufficient radiological or clinical response doxorubicin 50 mg m^–2^ was added to the regimen (*n*=1). Patients with DLBCL were treated with R-CHOP (rituximab 375 mg m^–2^, cyclophosphamide 750 mg m^–2^, vincristine 2 mg, doxorubicin 50 mg m^–2^ and prednisone 40 mg m^–2^ (for 5 days)) every 21 days.

### Circulating biomarkers

Serum was analysed for (a) nDNA using the Cell Death Detection ELISA (Roche, Basel, Switzerland), (b) CK18 using the M65 ELISA (Peviva, Sweden) and (c) FLT3 ligand using an ELISA (R&D, Abingdon, UK; #DFK00). All analyses followed the manufacturer's instructions and were conducted according to Good Clinical Laboratory Practice. As nDNA is a quasi-quantative ELISA, data are expressed as optical density readings. The M65 and FLT3 ligand ELISAs are semi-quantative assays and data are expressed in U l^–1^ and pg ml^–1^, respectively. Both full blood count and lactate dehydrogenase (LDH) were determined independently by the Christie Pathology Laboratories (Manchester, UK).

### Image analysis

X-ray computed tomography (CT) examination was performed before and after therapy. Patients were imaged on a LightSpeed Plus CT scanner (GE Medical Systems, Slough, UK) with typical clinical helical acquisition variables (tube voltage 120 kV, tube current 40 mA). Images were acquired following intravenous injection of 200 ml Omnipaque-140 (GE Medical Systems) and reformatted to produce contiguous slices with no overlap. Slice thickness was 5 mm in all but four cases, where initial data constraints necessitated a slice thickness of 7.5 mm. Nodal tumour burden was assessed by measuring enlarged lymph nodes. Two types of measurements were made based on (a) the sum of the bi-dimensional measurements (units cm^2^) as recommended in the Cheson criteria (Cheson *et al*, 2007) and (b) tumour volume, calculated as the product of the number of voxels and voxel volume (units mm^3^). Extra-nodal disease in solid organs and bone was excluded. All measurements were performed by a radiologist blinded to the serological biomarker data.

### Toxicity recording

Epithelial toxicity was assessed when the patient attended for their second cycle of chemotherapy. The maximum experience of vomiting, diarrhoea, stomatitis, inter-current infection and hepatotoxicity was graded according to the Common Terminology for Classification of Adverse Events (CTCAE) version 3.0 (Trotti *et al*, 2003) and individual grades summed to give an overall subjective toxicity score. Multifactorial symptoms of nausea and constipation were not included in the determination of the epithelial toxicity score, as it can be difficult to attribute these symptoms directly to epithelial damage. Nausea following chemotherapy may be initiated by cerebral signalling, and constipation result from changes to autonomic control of intestinal motility.

### Statistics

Statistical analysis was performed using GraphPad Prism version 5.02 for Windows, (GraphPad Software, San Diego, CA, USA, www.graphpad.com) and probability values of *P*⩽0.05 considered significant throughout. As the biomarkers were positively skewed, non-parametric tests were used to satisfy the assumptions of variance between sample groups. Differences between groups were tested using Mann–Whitney (M–W) *U*-test if comparing two groups or Kruskal–Wallis (K–W) (followed by Dunn's *post hoc* test if significant) if comparing more than two groups. Relationships were examined using the non-parametric Spearman's rho bivariate correlation with a two-tailed test for significance. The Wilcoxin signed test (WST) was used to assess whether changes in biomarkers following chemotherapy were significantly different from 0%. The area under the curve (AUC) was calculated using the trapezoid method. In the absence of pre-existing data, no formal statistical power calculations were performed.

## Results

### Utility of circulating nDNA as a biomarker in lymphoma

**Figure 2:**
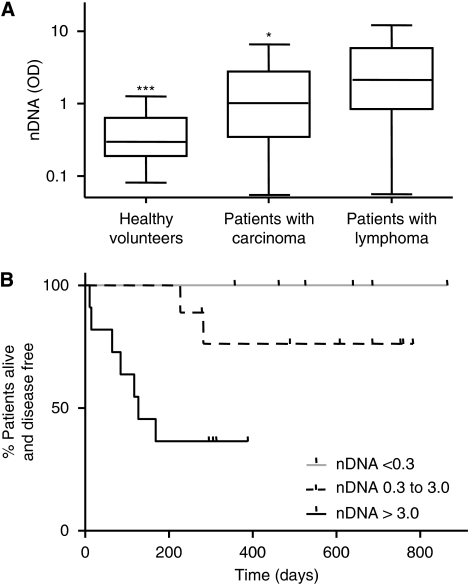
(**A**) Baseline levels of nDNA in patients with lymphoma compared with patients with carcinoma and healthy volunteers (^*^significantly different from patients with lymphoma *P*<0.05;. ^***^significantly different from patients with lymphoma *P*<0.001). (**B**) Prognostic impact of baseline nDNA in patients with diffuse large B-cell non-Hodgkin lymphoma shown by Kaplan–Meier curve for progression-free survival.

Baseline levels of nDNA were significantly higher in the 49 patients with lymphoma (geometric mean 1.58) compared with 61 healthy volunteers (geometric mean 0.33; K–W *P*<0.001) and 193 patients with metastatic carcinomas (small cell lung cancer (*n*=67), non-small cell lung cancer (*n*=35) or colorectal cancer (*n*=80) (geometric mean 0.94; K–W *P*<0.05 see Figure 2A). No statistically significant relationship was found between lymphoma histology and baseline nDNA levels (K–W *P*=0.99) although the patients with the four highest values all had DLBCL. No stage-related trend in baseline nDNA was identified, there was no difference between patients with/without ‘B’ symptoms (defined as weight loss >10% of baseline, drenching night sweats or unexplained fevers >38 °C; see Table 2) and no correlation with LDH levels was seen (*R*=0.26). Baseline levels of nDNA did not correlate with baseline nodal tumour burden using 2D or volumetric measurements.

### Prognostic significance of baseline circulating nDNA

In the DLBCL cohort, disease progression occurred in nine patients with a median time to progression of 115 days (range 10–277). The median follow-up in non-progressors was 517 days (range 274–851). Baseline nDNA levels were significantly higher in progressors (geometric mean 3.62, range 0.50–11.65) compared with non-progressors (geometric mean 1.0, range 0.06–21.5; *P*<0.05). No progression was seen in patients with nDNA levels below the median observed in healthy subjects (nDNA<0.3). In patients with levels above the maximum observed in healthy subjects (nDNA >2.9; *n*=11), the median PFS was 83 days (log-rank; *P*<0.001) and five patients died with a median OS of 63 days; *P*<0.05) (Figure 2B). There was no significant difference between baseline LDH levels in progressors/non-progressors with DLBCL (geometric mean 598 U ml^–1^ in progressors *vs* 681 U ml^–1^ in non-progressors (*P*=0.37).

In the patients with FL, 5 of 10 patients progressed with a median time to progression of 295 days (range 147–627) and a median PFS in the remainder of 707 days (range 493–8570). There was no difference between the baseline levels of nDNA in progressors (geometric mean 1.37) *vs* non-progressors (geometric mean 2.55).

Only one patient with HL progressed and subsequently died; prognostic outcome analysis was not therefore performed in this cohort.

### Changes in circulating nDNA following therapy

**Figure 3:**
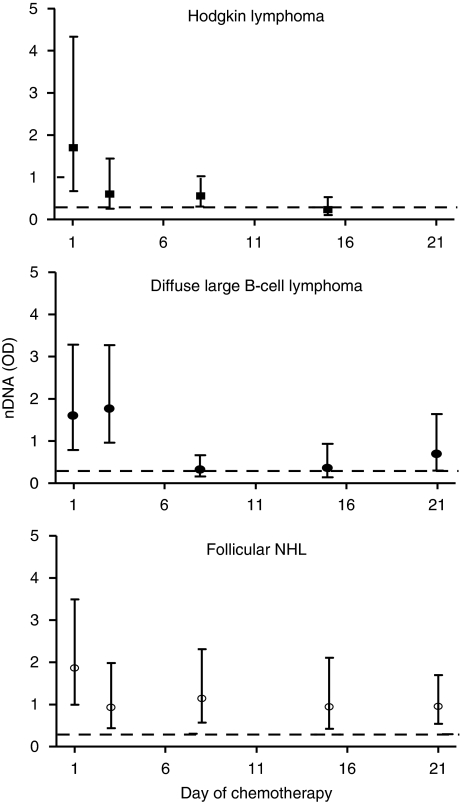
Levels of circulating nDNA in patients with lymphoma treated with chemotherapy by histological sub-type (samples evaluable at day 1, 3, 8 and 15 for all sub-types and day 22 for FL and DLBCL; dotted line represents mean value of nDNA in healthy subjects).

In patients with HL, nDNA levels had decreased by 48 h following therapy. By day 15, levels were similar to healthy controls (0.24 *vs* 0.29; M–W NS with mean change of −2.86, range −9.142 to 0.028; WST *P*<0.001) (Figure 3).

In patients with DLBCL, nDNA levels did not decrease until day 8 (Figure 3) (mean change −3.64, range −20.01 to 1.62; WST *P*<0.01). Nucleosomal DNA levels were lowest at this point, significantly lower than baseline and no longer significantly elevated compared with healthy controls (0.32 *vs* 0.29 M–W NS). Initial decreases in nDNA were observed following therapy in all patients with elevated levels, however, in those who subsequently progressed, significantly higher levels of nDNA (geometric means 1.58 *vs* 0.15; *P*<0.01) were seen by the end of therapy.

In patients with FL, nDNA levels changed more gradually during the first three weeks of treatment. Nucleosomal DNA levels were declining by day 3 (Figure 3), a decline sustained throughout the first cycle (mean change at day 22 −1.53 (range −8.73 to 1.49); WST *P*=0.23). However, in contrast to HL and DLBCL, these changes from baseline did not reach statistical significance and levels of nDNA at day 22 remained elevated compared with healthy controls (geometric means baseline 1.87; day 22 0.95; controls 0.29; K–W *P*<0.005). Patients with FL where the nDNA levels failed to fall during the first week of therapy had a significantly worse outcome with median PFS of 405 days, while only one patient with a falling nDNA at day 8 had progressed with a median follow-up of 658 days (*P*<0.05; Supplementary Figure 1).

Analyses were performed to assess whether early changes in nDNA levels following treatment could predict subsequent radiological response to therapy. Overall, 26 patients had evaluable CT examinations at baseline and after therapy (Supplementary Table 1). Levels of nDNA during the first week of therapy (assessed by the AUC days 1–8) correlated strongly with initial nodal tumour burden measured in both 2-D and volume (*R*=0.53 and 0.49, respectively; *P*<0.01), however, there was no statistically significant relationship between early changes in nDNA and changes in tumour volume on completion of therapy.

Taken together, these results suggest that circulating nDNA levels may have utility as a pharmacodynamic biomarker based on the rapid decreases in nDNA levels observed following therapy. Patients with DLBCL/FL and persistent elevation or rising levels in during therapy had a poorer outcome.

### Utility of circulating CK18 as an epithelial toxicity biomarker

Baseline levels of circulating CK18 in patients with lymphoma (geometric mean 348 U l^–1^) were no greater than observed in healthy subjects (geometric mean 347 U l^–1^) and were significantly lower than patients with epithelial cancers (geometric mean 712 U l^–1^, *P*<0.001).

Following therapy, CK18 increases peaked at day 3 (HL, mean change +33%, range −17 to 113% DLBCL mean change +33%, range −44 to 113%) and were recovering by day 15 (HL mean change 14%, range −6 to 64% DLBCL mean change +15%, range −52 to 84%). No significant elevation was seen in CK18 following chemotherapy in patients with FL (day 3 change +9%, range −27 to 39% and day 15 change −4%, range −52 to 26%), in keeping with the lower incidence of epithelial toxicity observed with this chemotherapy regimen.

**Figure 4:**
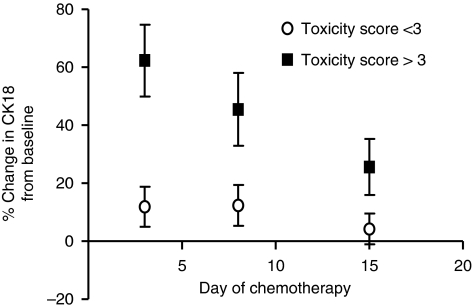
Changes in circulating CK18 following chemotherapy in patients with lymphoma according to CTCAE epithelial toxicity score.

In patients with more severe toxicities, higher and more durable peaks in CK18 were seen following therapy (Figure 4). The change in CK18 at day 3 gave the greatest power to discriminate patients experiencing the worst epithelial toxicity. The largest changes in CK18 were seen in patients with a CTCAE score >3 (mean increase in CK18 at day 3 for a toxicity score 0=+12% score 1=−7% score 2=+3% score 3=+38% score 4=+69% and score 5=+73%). This showed significant differences with a mean change in CK18 of 62% in patients with toxicity score ⩾3 (*n*=9) compared with 12% in patients with toxicity score <3 (*n*=37; M–W *P*<0.005) with an estimated AUC for the receiver operating characteristics (ROCs) curve to detect an epithelial toxicity CTCAE score of ⩾3 of 0.85 (*P*<0.005). These data suggest that CK18 can be used to predict epithelial toxicity in patients with lymphoma.

### Utility of FLT3 ligand as a biomarker of myelosuppression

FLT3 ligand was evaluable in 43 patients (10 HL, 10 FL and 23 DLBCL). Baseline levels were low for all three lymphoma types, although higher levels were seen in FL than DLBCL. There was no significant difference between patients with documented bone marrow involvement and those without (geometric mean involved 115 pg ml^–1^ (range 26–411 pg ml^–1^) *vs* uninvolved 90 pg ml^–1^ (range 29–229 pg ml^–1^)).

In patients with HL treated with AVBD, an elevation in FLT3 ligand was seen by day 3 (mean change +166%, range +46 to 356%), which resolved by day 15 (mean change −2%, range −40 to 36%). In patients with DLBCL, increases in FLT3 ligand did not occur until day 8 (mean change +261%, range 16 to 796%), and while in most was resolving by day 22, some patients had persistent elevations (mean change +92%, range −11 to 1280%).

In patients with FL, smaller elevations were seen compared with other lymphoma subtypes, in keeping with the lower myelosuppressive potential of R-CVP. As in DLBCL maximum elevation was seen at day 8 (mean change +57%, range 24–111%) with resolution by day 22 (mean change +3%, range −54 to 51%).

**Figure 5:**
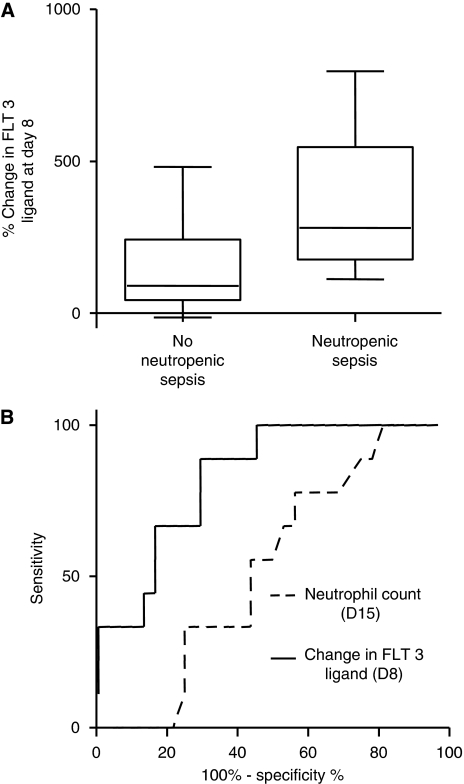
(**A**) Comparison of changes in FLT3 ligand at day 8 following chemotherapy between patients who did and did not experience neutropenic sepsis (NPS) at any point during treatment. (**B**) Receiver operating characteristic curve comparing the utility of FLT3 ligand measurement at day 8 to neutrophil count at day 15 to predict subsequent admissions with NPS.

The correlation of increases in FLT3 ligand at day 8 with myelosuppression was assessed in all 43 patients, and a significant correlation with neutropenia (assessed at day 15) was observed (*R*=−0.34; *P*<0.05). Nine patients (21%) developed neutropenic sepsis (NPS) at some point during treatment. These patients had significantly higher increases in FLT3 ligand at day 8 of their first cycle of chemotherapy (mean 349%, range 111 to 796%) than patients who did not develop NPS (mean 131%, range −13 to 482%), (Figure 5A). There was no difference between the nadir neutrophil count in the two groups (mean 5.9, range 0.3–24.4) in patients with NPS *vs* 4.3, range 0.2–13.9 in patients without NPS. Therefore, measurement of circulating FLT3 ligand at day 8 was a better predictor of a future episode of NPS then nadir neutrophil count routinely checked at day 15 (AUC for the ROC curves 0.83 (95% CI 0.70–0.97) for FLT3 ligand *vs* 0.53 (95% CI 0.34–0.72) for the neutrophil count, Figure 5B).

## Discussion

Improving the efficacy and reducing the toxicity of cancer treatment are amongst the biggest challenges facing oncologists. Developing readily measurable biomarkers to accurately predict and report these endpoints is therefore desirable. The study presents for the first time, a potential clinical role for a panel of circulating biomarkers in patients with lymphoma and suggests the need to further evaluate their utility.

In this study, pretreatment nDNA levels in DLBCL correlated strongly with PFS and overall survival. Prognosis in this disease can be estimated by determination of the revised-international prognostic index, which incorporates both clinical parameters and LDH (Sehn *et al*, 2007). This study was not designed or powered to assess the additional prognostic information given by determination of nDNA, however, the strong association with poor outcome in the absence of any observed association with traditional prognostic parameters (e.g. tumour stage and B symptoms), suggests a putative role for nDNA as a new independent prognostic biomarker. This should now be assessed in larger cohorts.

The use of nDNA as a PD biomarker following therapy may give the most additional clinical utility. In all lymphoma sub-types rapid falls in nDNA were seen following the initiation of therapy. Notably, in FL, persistent elevations in nDNA during the first week of treatment were associated with a significantly shorter time to progression. These findings suggest that nDNA could be used both to guide early changes in therapy if an insufficient biomarker response is observed, and also allow comparison between different regimens or dosing schedules in early clinical trials. Although other cell death products have been assessed as PD biomarkers in lymphoma such as caspase 3 and cytochrome *c* (Barczyk *et al*, 2005; Deligezer *et al*, 2006), nDNA has the advantage that extensive validation and qualification of this assay has already been performed in other tumour types (Holdenrieder *et al*, 2008).

As nDNA is a quasi-quantitative assay (Cummings *et al*, 2008), it is difficult to directly compare results between laboratories. Results in healthy volunteers are consistently and reproducibly low (Hou *et al*, 2009). The average values derived in a large cohort of healthy controls offers a surrogate measure so that other laboratories can indirectly compare their results with those presented here.

Prediction of toxicity may allow pre-emptive action such as early clinical review and the potential use of supportive care medication. We did not examine a detailed temporal relationship between a rise in CK18 levels and the appearance of epithelial toxicity. Instead, a maximum peak in CK18 was recorded at day 3 and an obvious difference in CK18 profiles between patients with and without subsequent toxicity was observed before the clinical manifestation of toxicity appeared (which typically occurred at days 7–14). Strategies that increase the dose intensity of chemotherapy would be facilitated by a measure of epithelial toxicity and the potential of this approach is currently being tested in patients with HL treated in a phase I dose-escalation study.

Other biomarkers of small bowel toxicity have been evaluated including carbon^13^-sucrose breath tests, plasma citrulline and faecal calprotectin and lactoferrin (Lutgens *et al*, 2005; Hille *et al*, 2009). However, the clinical utility of these biomarkers has yet to be fully determined. In addition, CK18 gives a global measure of epithelial damage, as compared with the specificity to small bowel damage of the assays described above.

The mechanism of increasing FLT3 ligand in response to bone marrow stress remains unclear but local release in the bone marrow, potentially by the stroma, is the leading hypothesis (Prat *et al*, 2005). In mice, circulating FLT3 ligand levels at day 3 following radiotherapy predicted the length and depth of subsequent pancytopenia (Prat *et al*, 2006). In humans, FLT3 ligand levels rose within 24 h of radiotherapy and before the effect on peripheral blood counts (Bertho *et al*, 2001). Similarly, in this study the rise in FLT3 Ligand was observed before the neutrophil nadir.

An observed increase in FLT3 ligand at day 8 in cycle 1 would potentially allow the use of growth factors or antibiotics to prevent NPS, even during this cycle of therapy. However, it may be particularly useful to allow interventions in patients who first experience NPS in later cycles of therapy (four patients in this study) or have repeated episodes of NPS (1 patient). As CK18 and FLT3 ligand appear to measure host toxicity objectively, potentially they could be used to adjust doses in an individual patient in order to maximise the therapeutic index, or in early clinical trials to assess the potential for future problematic toxicity. The different chemotherapy regimens examined in this study demonstrated that these biomarkers are useful in predicting the patients who will experience side effects both from therapies where relatively high rates of toxicity can be observed (e.g., ABVD and R-CHOP), but also from better tolerated regimens (e.g., R-CVP). The utility of these biomarkers in patients receiving mechanism-based agents is now being examined.

Our results show the potential utility of a panel of validated biomarker assays in patients with three sub-types of lymphoma. The data suggest first that baseline nDNA in DLBCL gives additional prognostic information over LDH and early changes during therapy may predict subsequent outcome in FL, second, that rises in CK18 on chemotherapy give early warning of epithelial toxicity and third, that substantial elevation in FLT3 ligand identifies patients at risk of NPS. These results argue for inclusion of these biomarkers in larger studies testing individualised treatment strategies.

## Figures and Tables

**Table 1:** Clinical characteristics of patients with Hodgkin lymphoma, follicular lymphoma and diffuse large B-cell lymphoma

	**HL**	**FL**	**DLBCL**
Number	13	10	26
Age (median (range))	38 (18–61)	60 (47–85)	69 (22–90)
Gender male: female (% male)	4:9 (31%)	5:5 (50%)	14:12 (54%)
			
*PS*			
0	5 (38%)	4 (40%)	4 (15%)
1	7 (54%)	6 (60%)	16 (62%)
2	1(8%)	0	2 (8%)
3	0	0	4 (15%)
			
*Stage*			
I	1 (8%)	0	4 (15%)
II	6 (46%)	2 (20%)	7 (27%)
III	2 (15%)	2 (20%)	3 (11%)
IV	4 (31%)	6 (60%)	12 (46%)
‘B’ symptoms	7 (54%)	0	13 (50%)
Therapy	ABVD ( × 3) 1	R-CVP 10 (two converted to R-CHOP)	R-CHOP 24 (two converted to R-GCVP)
	ABVD ( × 6) 12		R-GCVP 2

Abbreviations: ABVD=therapy with doxorubicin, bleomycin, vinblastine and dacarbazine; ‘B’ symptoms=weight loss >10%, drenching night sweats or unexplained fevers >38 °C; DLBCL=diffuse large B-cell lymphoma; FL=follicular lymphoma; HL=Hodgkin lymphoma; PS=performance status; stage=as defined by Ann-Arbor Staging System; R-CVP=therapy with rituximab, cyclophosphamide, vincristine and prednisone; R-CHOP=therapy with rituximab, cyclophosphamide, doxorubicin, vincristine and prednisone; R-GCVP=therapy with rituximab, gemcitabine, cyclophosphamide, vincristine and prednisone.

**Table 2:** Baseline levels of circulating biomarkers in patients with lymphoma by histology and stage

	**LDH (U ml^−1^)**	**nDNA (OD)**	**CK18 (U l^−1^)**	**FLT3 ligand (pg ml^−1^)**
*Histology*				
Hodgkin lymphoma	403 (236–621)	1.59 (0.06–9.62)	267 (126–470)	96 (40–229)
Follicular lymphoma	404 (301–699)	1.87 (0.57–10.90)	331 (220–580)	153 (90–411)
Diffuse large B-cell lymphoma	626 (310–4030)	1.60 (0.06–21.5)	405 (192–1982)	85 (26–181)
				
*Stage*				
I	448 (310–653)	2.05 (0.21–8.91)	301 (194–472)	94 (55–134)
II	406 (236–915)	1.56 (0.22–19.05)	338 (193–665)	86 (29–162)
III	650 (360–1331)	2.35 (0.62–21.5)	299 (126–571)	132 (74–229)
IV	555 (270–4030)	1.42 (0.05–11.65)	390 (192–1982)	105 (26–411)
A	454 (236–1311)	1.66 (0.06–21.5)	325 (192–580)	112 (47–411)
B	605 (288–4030)	1.64 (0.06–19.05)	386 (126–1982)	88 (26–229)

Abbreviations: CK18=cytokeratin 18; LDH=lactate dehydrogenase; nDNA=nucleosomal DNA.
