# Supplementary figures and images for: Assessment of circulating biomarkers for potential pharmacodynamic utility in patients with lymphoma
Source: Br J Cancer. 2011 Jan 18;104(4):719–25. doi: 10.1038/sj.bjc.6606082 (PMC3049589; doi:10.1038/sj.bjc.6606082)

## Slide 1
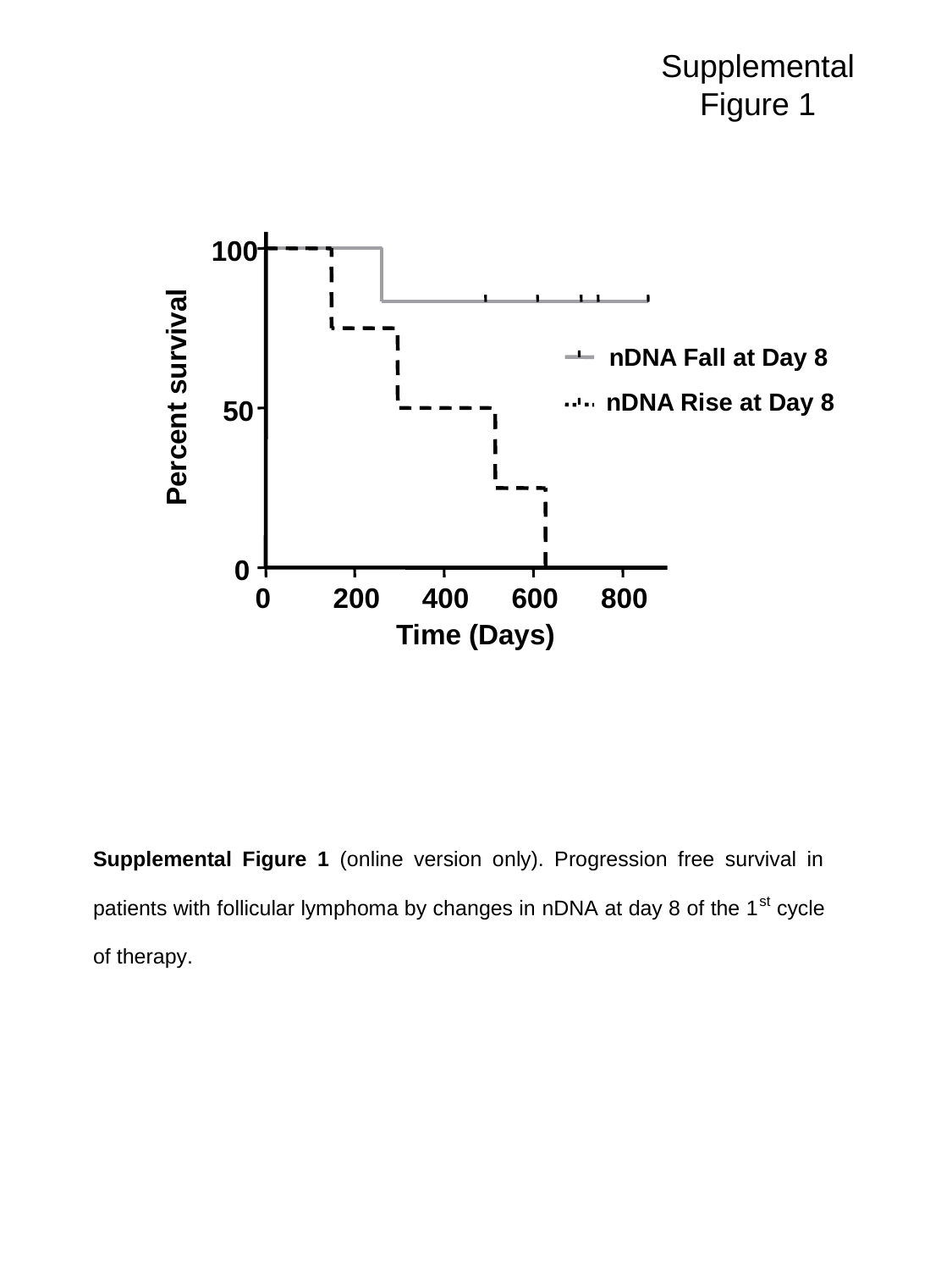

Supplemental Figure 1
100
nDNA Fall at Day 8
nDNA Rise at Day 8
Percent survival
50
0
0
200
400
600
800
Time (Days)

Supplement: Supplementary Figure 1 [file 6606082x1.ppt]
